# The influence of endophytes on rice fitness under environmental stresses

**DOI:** 10.1007/s11103-021-01219-8

**Published:** 2021-12-02

**Authors:** Showkat Ahmad Ganie, Javaid Akhter Bhat, Alessandra Devoto

**Affiliations:** 1 Plant Molecular Science and Centre of Systems and Synthetic Biology, Department of Biological Sciences, Royal Holloway University of London, Egham, Surrey TW20 0EX UK; 2 State Key Laboratory for Crop Genetics and Germplasm Enhancement, College of Agriculture, Nanjing Agricultural University, Nanjing, 210095 China

**Keywords:** Abiotic/biotic stress, Endophyte, Microbiome, *Oryza sativa*, Rice, Salinity, Systemic resistance

## Abstract

**Key Message:**

Endophytes are crucial for the promotion of rice growth and stress tolerance and can be used to increase rice crop yield. Endophytes can thus be exploited in biotechnology and genetic engineering as eco-friendly and cost-effective means for the development of high-yielding and stress-tolerant rice plants.

**Abstract:**

Rice (*Oryza sativa*) crop is continuously subjected to biotic and abiotic stresses, compromising growth and consequently yield. The situation is exacerbated by climate change impacting on ecosystems and biodiversity. Genetic engineering has been used to develop stress-tolerant rice, alongside physical and chemical methods to mitigate the effect of these stresses. However, the success of these strategies has been hindered by short-lived field success and public concern on adverse effects associated. The limited success in the field of stress-tolerant cultivars developed through breeding or transgenic approaches is due to the complex nature of stress tolerance as well as to the resistance breakdown caused by accelerated evolution of pathogens. It is therefore necessary to develop novel and acceptable strategies to enhance rice stress tolerance and durable resistance and consequently improve yield. In the last decade, plant growth promoting (PGP) microbes, especially endophytes, have drawn the attention of agricultural scientists worldwide, due to their ability to mitigate environmental stresses in crops, without causing adverse effects. Increasing evidence indicates that endophytes effectively confer fitness benefits also to rice under biotic and abiotic stress conditions. Endophyte-produced metabolites can control the expression of stress-responsive genes and improve the physiological performance and growth of rice plants. This review highlights the current evidence available for PGP microbe-promoted tolerance of rice to abiotic stresses such as salinity and drought and to biotic ones, with special emphasis on endophytes. Associated molecular mechanisms are illustrated, and prospects for sustainable rice production also in the light of the impending climate change, discussed.

## Introduction

Rice (*Oryza sativa* L.) is the major cereal crop which provides staple food for more than half of the world’s population (Ganie et al. 2014). The global rice utilization in 2021/2022 is expected to be 520.8 million tonnes, which is up by 1.5% from the previous season (FAO 2021). In view of the alarmingly growing human population, the demand for rice is predicted to rise further. However, rice crop is highly susceptible to biotic and abiotic stresses, leading to significant losses in grain yield and quality (Cohen and Leach 2019; Ganie et al. 2020; Muthu et al. 2020; Salgotra et al. 2021), which is expected to worsen due to the impact of climate change. Annually, abiotic and biotic stresses cause almost 50% and 15% loss, respectively, in global rice production (Maxmen 2013; Anami et al. 2020). The rise in the temperature and precipitation will alter the geographic distribution and host range of various pests and pathogens, aggravating further rice productivity (Johnson 2011). It is therefore necessary to develop new strategies to enhance rice stress tolerance and consequently yield.

Environmental stresses impair agronomic and yield-related traits of rice, including flowering time, maturity time, spikelet fertility, grain filling, weight and quality (Kamoshita et al. 2004; Das and Rao 2015; Ganie et al. 2019; Muthu et al. 2020). Stresses negatively affect several important growth-related physiological and metabolic processes such as chlorophyll biosynthesis, photosynthesis, transpiration, cell wall metabolism, hormonal balance, seed germination and nutrient acquisition (Rizwan et al. 2016; Pandey et al. 2017; Cohen and Leach 2019; Ganie and Ahammed 2021). To avoid stress-induced adverse effects, rice plants undergo rapid morphological, physio-biochemical and molecular changes (Singh et al. 2016; Formentin et al. 2018; Huang et al. 2020; Muthu et al. 2020; Ganie et al. 2021). For example, level of phytohormones and phytohormone-mediated signalling pathways play a crucial role in rice survival under stress conditions (Yang et al. 2013; Sah et al. 2016). These pathways modulate physio-biochemical and molecular stress tolerance processes, such as activation of antioxidant defence system, redox homeostasis, enhanced photosynthesis efficiency, chlorophyll biosynthesis, nutrient balance, and activation of stress-related gene expression (Yang et al. 2013; Formentin et al. 2018; Faizan et al. 2021). The concentration of abscisic acid (ABA) increases considerably in rice under various stress conditions such as drought and salt, leading to stomatal closure and activation of stress-responsive genes encoding transcription factors (Ye et al. 2012; Formentin et al. 2018). ABA regulates the expression of stress-responsive transcription factors such as ABA-responsive element (ABRE)-binding protein/ABRE-binding factor (AREB/ABF), APETALA2/ethylene responsive factor (AP2/ERF), NAM-no apical meristem, Petunia+ATAF1–2-*Arabidopsis thaliana* activating factor+CUC2-cup-shaped cotyledon, Arabidopsis (NAC; NAM, ATAF, and CUC), basic leucine zipper (bZIP), myelocytomatosis oncogenes (MYC), and myeloblastosis (MYB) (Singh and Laxmi 2015). These transcription factors in turn modulate the expression of other stress-responsive genes encoding antioxidative enzymes, molecular chaperones, ion-channel proteins, osmo-protectants (Ganie et al. 2019). Hence, the elucidation of the mechanisms regulating rice stress response at physiological, biochemical and molecular levels will assist to develop stress-tolerant rice cultivars.

Organic and inorganic fertilizers as well as fungicides and pesticides, are being increasingly used to boost productivity and stress tolerance of rice crop (Gribaldi et al. 2017; Muhammad et al. 2019; Iqbal et al. 2020; Zhang et al. 2020). However, besides being costly the application of these chemicals reduces the quality of soil and causes environmental pollution. To this end, the development of high-yielding and stress tolerant crop varieties, without the need to use chemical treatments, will reduce the impact on climate change (Bhat et al. 2020). In the last four decades, approaches including genetic modification, mutational selection and breeding traits from wild plants, have been extensively used to improve cultivars of many crop species, including rice, that can adapt to and survive in high stress habitats (reviewed by Ganie et al. 2021; Menguer et al. 2017; Qaim 2020). However, the success of these approaches under field conditions has been quite variable, due to the genetic complexity of stress tolerance processes and additional environmental pressure (Wei et al. 2020). More recently, greatest consideration has been given to the use of the microbiome for devising innovative methods to improve rice stress responses.

Plants are closely associated with huge masses of microbes in rhizosphere (microbes in soil surrounding roots), endosphere (microbes within roots), leaves (phyllosphere), as well as pollen, nectaries, all together representing the microbiome (Liu et al. 2017a; Álvarez-Pérez et al. 2019; Liu and Brettell 2019; Liu et al. 2020a; Sheteiwy et al. 2021a). The communities of microbes hosted by plants are not static; their size, structure and functions are affected by environmental stresses (Liu et al. 2020a). Coevolution of plants and microbes during time has affected microbiome changes (Durán et al. 2018; Chen et al. 2018; Kwak et al. 2018; Liu et al. 2020a). Plants under stress conditions can benefit from the presence of microbes to survive by adopting the strategy of ‘cry-for-help’, whereby plants produce ‘chemical messengers’ to activate beneficial microbial endophytes to alleviate the stress-induced damage (Liu et al. 2020a). In general, the plant-endophyte interaction is very important for the acquisition of nutrients, growth, development and enhancing tolerance to different environmental stresses.

Endophytes include both bacteria and fungi that colonize the plant tissues either intracellularly or intercellularly without causing any harm to the plant (Wilson 1995). Endophytes have been reported from almost all plant species, forming either obligate or facultative associations. The relationship of endophytes with their hosts can be mutualistic or antagonistic (Nair and Padmavathy 2014). Endophytic growth is strictly regulated by plants (Dudeja et al. 2012). Stable symbiosis between endophytes and plants includes the production of compounds by the former that positively influence plant growth survival under unfavourable conditions (Lee et al. 2004; Das and Varma 2009; Sheteiwy et al. 2021b). The production of phytohormones, exopolysaccharides (EPS), enzymes like 1-aminocyclopropane-1-carboxylic acid deaminase (ACCD), nitrogen fixation and improving the availability of mineral elements are some of the traits/mechanisms involved in promoting plant growth (Eid et al. 2021). Furthermore, the endophytes play a critical role in the biocontrol of phytopathogens as they colonize the same ecological niche (Eid et al. 2021). Biocontrol mechanisms include the production of antifungal or antibacterial agents, siderophores, nutrient competition, and the induction of systemic-acquired host resistance or immunity (Thakur et al. 2020). Endophytes can also confer metal resistance to plants and reduce metal toxicity due to their intrinsic ability to tolerate different types of metals (Ma et al. 2016).

In rice, various types of endophytes have been isolated and characterized for their role in promoting rice health and vigour under biotic and abiotic stress conditions. The beneficial effects of endophytes on rice growth are mediated by several functional traits such as phytohormones and siderophores production, nitrogen fixation and metal detoxification (Potshangbam et al. 2017; Kumar et al. 2020). However, it is still unclear whether specificity of interaction exists between endophytes and rice.

This review provides an account of the current knowledge on the role of microbes, with a particular focus on endophytes and their effect on rice growth, development and yield under stress-free and stress conditions, and on the molecules and the mechanisms underlying these processes. The review also discusses the potential contribution of endophytes towards rice improvement for a more sustainable future.

## Regulation of rice growth by endophytes under non-stress conditions

Endophytes can influence rice growth, biomass production and yield under normal (abiotic or biotic stress-free) conditions. They promote rice growth through phytohormones (such as auxins and gibberellins, GAs), siderophores (scavenging ferric iron and other metal element from the environment), solubilization of inorganic phosphate, fixation of nitrogen, and suppression of ethylene levels via production of ACCD, as illustrated below and summarized in Table 1. Whether these growth enhancing microbes form an active interaction with rice is however poorly understood (Singha et al. 2021). Several studies have demonstrated the role of endophyte-produced phytohormones, namely gibberellic acids, auxins and cytokinins on rice growth. For instance, the fungus *Cladosporium sphaerospermum* produces gibberellins (GA7 and GA4), and the inoculation of this endophyte enhances rice biomass (Hamayun et al. 2009). Inoculation of plants with key growth regulators like indole acetic acid (IAA)-producing endophytic bacterium *Burkholderia vietnamiensis* improves rice growth and yield (Trân Van et al. 2000). IAA-producing endophytic fungal isolates from aromatic rice, positively regulate rice seed germination (Syamsia Kuswinanti et al. 2015). Similarly, IAA-producing bacterial endophytes such as *Micrococcus yunnanensis* RWL-2, *Micrococcus luteus* RWL-3, *Enterobacter soli* RWL-4, *Leclercia adecarboxylata* RWL-5, *Pantoea dispersa* RWL-6, and *Staphylococcus epidermidis* RWL-7, were reported to promote rice shoot and root elongation, biomass production and chlorophyll content (Shahzad et al. 2017a). Likewise, the IAA-producing soil yeast *Candida tropicalis* HY (CtHY) improves rice seedlings root growth (Amprayn et al. 2012). IAA- and GA-producing *P. glomerata* and *P. formosus* fungal endophytes also substantially enhance chlorophyll content, shoot length and biomass in rice (Waqas et al. 2014).

**Table 1:** Effect of different endophytes on rice physiology and growth under non-stress conditions

Endophytes	Action on molecular and physiological mechanisms	Rice traits improved	Reference
*Burkholderia vietnamiensis*	Produces siderophores and IAA	Shoot and root biomass, leaf surface, tiller number and seed yield	Trân Van et al. (2000)
*Micrococcus* spp., *Enterobacter soli*, *Leclercia adecarboxylata*, *Pantoea dispersa*, *Staphylococcus epidermidis*	Produce growth promoting hormones viz., indole acetic acid (IAA)	Shoot and root, seedlings biomass, chlorophyll content	Shahzad et al. (2017a)
*Lysinibacillus sphaericus*	Produces 1-aminocyclopropane-1-carboxylate deaminase (ACCD) and modulates the ethylene levels in the plant	Number of panicles and grain per plant, straw and grain dry weight; nitrogen and phosphorus uptake	Shabanamol et al. (2020)
*Pseudomonas* sp., *Achromobacter* sp., *Burkholderia* sp., *Klebsiella pneumoniae*	Produce ACCD and modulate ethylene levels in the plant	Shoot and root length, fresh and dry weight, yield	Blaha et al. (2006)
*Pseudomonas koreensis*, *Arthrobactersp nitroguajacolicus*, *Klebsiella oxytoca*	Produce IAA and solubilize phosphorus for plant uptake	Shoot and root length, fresh and dry weight and phosphate uptake	Gusain et al. (2015)
*Azospirillum lipoferum*	Enhances phosphorus solubilization for plant uptake	Root length and overall surface area, shoot and root fresh and dry weight, leaf number and leaf and shoot length	Murty and Ladha (1988)
*Streptomyces* sp.	Produces siderophores and increases iron-uptake	Shoot and root length, fresh and dry weight	Rungin et al. (2012)
*Bacillus testaceum*, *Bacillus barbaricus*, *Bacillus subtilis*	Produce siderophore production and increase uptake of Iron	Shoot height, tillers number, flag leaf length, panicles and seeds number per plant, dry biomass of the whole plant and yield	Borah et al. (2018)
*Burkholderia vietnamiensis*	Nitrogen-fixation	Root length, shoot and root fresh and dry weight, yield	Puri et al. (2018)
*Pantoea agglomerans*	Nitrogen-fixation	Shoot and root fresh weight, flag leaf length, total plant biomass	Feng et al. (2006)
*Cladosporium sphaerospermum*	Produces phytohormones such as gibberellins (GA3, GA4, GA7)	Shoot length, leaf area, chlorophyll content, biomass and transpiration	Hamayun et al. (2009)
*Bacillus* sp.	Produces IAA and increases solubilization of phosphorus	Shoot and root length, total biomass and yield	Syamsia Kuswinanti et al. (2015)
*Candida tropicalis*	Produces IAA	Shoot and root dry weight, shoot and root length and seed germination rate	Amprayn et al. (2012)
*Phoma glomerata*, *Paecilomyces formosus*	Produce IAA and GA	Plant height, fresh and dry weight, and chlorophyll content	Waqas et al. (2014)

The plant gaseous hormone ethylene can inhibit rice growth and yield (Liu et al. 2008; Ma et al. 2014; Qin et al. 2019a). Endophyte-produced ACCD was shown to enhance rice growth in the absence of stress while decreasing ethylene levels in the plant (Wisniewski-Dyé et al. 2011; Kwak et al. 2012). For example, the production of ACCD by *Lysinibacillus*, leads to the conversion of aminocyclopropane carboxylic acid (ACC; precursor for ethylene biosynthesis) to ammonia and *alpha*-ketobutyrate, making ACC unavailable for ethylene biosynthesis (Santoyo et al. 2016). Other ACCD-producing endophytes, such as *Pseudomonas* sp., *Achromobacter, Burkholderia* sp., and *Klebsiella pneumoniae*, also inhibit ethylene biosynthesis in rice plants through this mechanism (Karthikeyan et al. 2012; Etesami et al. 2014; Santoyo et al. 2016).

Phosphorus (P) is the 2nd most essential nutrient for plant growth. Although abundant in soil, it is mostly unavailable to plants (Miller et al. 2010). In soil, P usually exists as mineral salts or in association with organic compounds (Miller et al. 2010). Bacterial species present in rhizosphere can solubilize the inorganic phosphate or secret the organic phosphates, making P available for plant absorption (Khan et al. 2010; El-Sawah et al. 2021). The liberation of organic phosphates requires the activity of enzymes such as phosphonatases, phytases and C–P lyases (Oteino et al. 2015). The solubilization of mineral phosphate is based on release of acid phosphatases and organic acids by endophytes (Illmer and Schinner 1995). Several studies have suggested that the use of phosphate solubilizing microbes has reduced the need of inorganic P fertilizers by 50%, without compromising yield (Jilani et al. 2007; Yazdani et al. 2009). Gusain et al. (2015) showed that inoculation with phosphate solubilizing *Pseudomonas* sp. considerably enhances phosphate uptake and growth in rice. Inoculation of rice seeds with *Azospirillum lipoferum* strain 34 H also has positive effects on phosphate solubilization, root and shoot length, as well as biomass in rice (Murty and Ladha 1988). In a similar fashion, PGPR isolate PGT3, increases seed germination, growth and biomass due to its ability to solubilize P in the rhizosphere, making it available for uptake.

Iron (Fe) is an essential micro-nutrient for the plant growth, serving as a co-factor for multiple enzymes such as those involved in N-fixation (Dhaliwal et al. 2019). Most of the Fe in soil is present in the insoluble ferric (Fe^3+^) form, as oxides, carbonates and hydroxides, not available for plant uptake (Ma et al. 2016). Microbes can secrete siderophores (low molecular weight compounds) under Fe deficient conditions, which bind with Fe^3+^, Fe^2+^, and other divalent metal ions (Saha et al. 2016), facilitating the uptake by plants hence benefitting growth (Eid et al. 2021; Beneduzi et al. 2012). It has been shown that wild-type strains of *Streptomyces* sp GMKU 3100 enhance rice growth through siderophore production (Rungin et al. 2012). Borah et al. (2018) demonstrated that siderophore producing endophyte bacteria such as *Bacillus testaceum*, *Bacillus barbaricus* and *Bacillus subtilis* promoted growth and increased yield by more than 3-fold in rice, halving application rates of chemical fertilizers.

Nitrogen (N) is an essential nutrient for rice growth, thus rice production relies heavily on inorganic N fertilizers (Dawe et al. 2000). Hence, in planta, improvement of N-fixation is an eco-friendly and cost-effective approach to prevent the excessive use of environmentally unsafe inorganic N fertilizers. Diazotrophs are bacteria capable of N-fixation by converting dinitrogen (N_2_) to ammonia (NH_3_) (Dilworth 1974). Several diazotrophic bacteria, including *Klebsiella oxytoca*, *Enterobacter cloacae*, *Alcaligenes*, and *Azospirillum*, have been isolated from rice rhizosphere (Elbeltagy et al. 2001; Yoneyama et al. 2017). Endophytic diazotrophs, such as *Acetobacter* spp., *Azoarcus* spp., and *Herbaspirillum* spp., occurring in gramineous, including rice, can also fix N (Reinhold-Hurek and Hurek 1998). *Azoarcus* sp. showed enhanced expression of nitrogen fixation (*nif*) genes, leading to the accumulation of nitrogenase in rice roots, thus suggesting their role in N-fixation (Reinhold-Hurek and Hurek 1998). Recently, Shabanamol et al. (2020) specifically reported on the positive effect of the inoculation with N-fixing endophytic diazotrophic bacterium *Lysinibacillus sphaericus* on rice yield and nutrient uptake. Moreover, the inoculation with N-fixing endophyte *Burkholderia vietnamiensis* strain AR1122 enhances rice grain yield and biomass (Puri et al. 2018), supporting its use as biofertilizer for sustainable rice crop production. Feng et al. (2006) also reported that rice seedlings inoculated with *Pantoea agglomerans* YS19 maintained a vigorous growth compared to non-inoculated ones.

Altogether, these studies demonstrate that endophyte-mediated production of phytohormones positively regulate rice growth, having seemingly specific effects on the growth of different organs. It is also evident that these microorganisms provide great potential to limit the use of P and N fertilizers.

## Effects of abiotic stress and plant genotype on rice endophytic community

The structure and diversity of rice endophytic communities are shaped by factors such as plant genotype, stress condition, and endophyte-host-pathogen interaction. For example, Walitang et al. (2018) showed that the endophytic communities of rice seeds are affected by the salt tolerance ability of different cultivars under salinity stress. Dominance and diversity of the bacterial community considerably differ between salt-sensitive and salt-tolerant rice cultivars, especially for bacterial classes such as *Microbacterium*, *Pantoea*, *Flavobacterium*, *Enterobacter* and *Curtobacterium* (Walitang et al. 2018). It has been suggested that drought-mediated changes in the rice microbiota aid the ability of the plant to withstand harsh environmental challenges (Santos-Medellín et al. 2017). Flooding or P stress also cause a strong shift in the rice diazotrophic endophytic bacterial community (Ferrando and Fernández Scavino 2015; Hameed et al. 2015). Lian et al. (2020) have shown that genetic variations in *SST* (*Seedling Salt Tolerant*) gene can influence the bacterial community under salt stress in the rice rhizosphere by controlling the secretion of metabolites. Management of soil microbiota is therefore a powerful tool to enhance rice stress tolerance.

Composition and diversity of endophytic microbes in rice are affected also by the physiological stages of development. Compared to its young stage, the mature developmental stage of black scented rice (known for its unique feature of antioxidant content), harbors higher endophytic abundance which has been associated with improved levels of antioxidant activity, suggesting that endophytes may contribute towards the higher antioxidant activity in mature black rice (Singha et al. 2021). Colonization efficiency of endophytic microbes changes therefore across the rice development, and it is possible that rice selects for specific group of endophytes based on the requirements of different growth stages. Hence, exploring developmental stage-specific native endophytic communities will prove very useful to improve biofertilizers to enhance stress tolerance at different growth stages (Ferrando and Fernández Scavino 2015).

## The beneficial effects of endophytes on growth and physio-biochemical traits of rice under abiotic stress conditions

Although rice has developed intricate physiological and biochemical mechanisms to overcome the adverse effects of abiotic stresses (Singh et al. 2014; Zafar et al. 2018; Ganie et al. 2019; Moraes de Freitas et al. 2019; Oladosu et al. 2019), endophytes can be used to improve its responses. Phytohormone-producing endophytes improve plant performance (see above) and reduce abiotic stress-induced damage in rice. For example, phytohormones like IAA and secondary metabolites (oxalic-, quinic-, tartaric-, and malic- acid) produced by the fungus *Paecilomyces formosus* LWL1 can mitigate heat stress in rice, promoting height, dry weight, and chlorophyll content, and reducing the endogenous levels of stress hormones like ABA and jasmonates (JAs) (Waqas et al. 2015). The acidification of the rhizosphere soil contributes to P availability for plant growth (Macías et al. 2020). Several endophytic bacterial isolates also produce phytohormones such as IAA, GAs and other organic acids to enhance rice growth under salt stress by downregulating endogenous ABA levels and increasing glutathione (GSH) and sugar content (Khan et al. 2020). The reactive oxygen species (ROS) scavenging potential of GSH may contribute to enhancing photosynthesis, and consequently sugars acting as osmolytes counteracting salt toxicity.

The GA-producing endophytic *Bacillus amyloliquefaciens* RWL-1 enhances growth, photosynthesis and biomass of rice seedlings subjected to Cu stress and ameliorates the plant stress response by regulating Cu uptake, carbohydrate, and amino acid levels, and antioxidation (Shahzad et al. 2019). Reduction of ABA and JA levels in the inoculated plants under Cu stress has also been observed. The same endophyte can promote the increase of salicylic acid (SA) and essential amino levels during salt stress to improve rice growth (Shahzad et al. 2017b). Altogether, these findings indicate that the reduction of endogenous stress-responsive hormones, such as the senescence promoting ABA (Song et al. 2016) and the growth-inhibiting JAs (Pérez-Salamó et al. 2019; Wang et al. 2020), represents a crucial mechanism employed by phytohormone-producing endophytes to mitigate different stress responses in rice. Maintenance of low endogenous levels of stress hormones (such as JAs) in the endophytes-inoculated plants may also contribute to priming to prepare the plants for subsequent challenges (Pieterse et al. 2000). Further, the reduction in the accumulation of α-linolenic acid, a substrate for JAs synthesis, derived from membrane lipids through the action of stress-induced lipases, in endophyte-inoculated and stressed rice (Shahzad et al. 2019), may be associated with the beneficial effect of phytohormone-producing endophytes on cell membranes. Incidentally, a reduction in stress-induced membrane damage in endophyte-inoculated rice, mirrored by lower malondialdehyde (MDA) content, has been reported (Li et al. 2012; Kakar et al. 2016; Jaemsaeng et al. 2018; Qin et al. 2019b; Shahzad et al. 2019; Sun et al. 2020; Tsai et al. 2020).

Nonetheless, variable effects on plant stress hormones have been observed. High ABA levels have been associated with reduced water-deficit in endophyte- inoculated rice. ABA-producing Salicaceae endophytes reduce stomatal conductance, density and leaf water potential, enhancing water use efficiency (WUE) under drought conditions (Rho et al. 2018).

Increased ROS production is a common manifestation of all stresses caused by stress-induced imbalances in different metabolic processes (Das and Roychoudhury 2014). Phytohormone-producing endophytes mitigate responses to different abiotic stresses in rice also by augmenting its antioxidant defence. For example, IAA-producing Class 2 endophytes confer habitat-adapted-symbiosis and improve drought and salt tolerance in part by reducing photobleaching and enhancing ROS scavenging (Redman et al. 2011). Similarly, the endophytic fungi from upland rice mediate rice drought tolerance by detoxifying ROS through increased superoxide dismutase (SOD), peroxidase (POD) and catalase (CAT) activities in the inoculated seedlings (Pang et al. 2020). In another case, rice plants treated with a consortium of two rhizobacteria (*Bacillus amyloliquefaciens* and *Brevibacillus laterosporus*) and biochemical growth elicitors (SA and β-aminobutyric acid) exhibited improved growth and survival, and showed reduced levels of chlorosis, leaf rolling, wilting and necrosis under cold and drought stress (Kakar et al. 2016). The underlying mechanisms included the reduction of electrolyte leakage and MDA content, and high amounts of proline, chlorophyll, and antioxidant enzymes. *Bacillus pumilus* also improves growth performance, chlorophyll and carotenoid content, and antioxidant-mediated defence response in inoculated rice during high salt stress (Kumar et al. 2021). These studies indicate that fungal and bacterial endophytes act as modulators of antioxidant enzyme activities, and metabolic pathways under abiotic stress conditions, to mitigate the oxidative stress-imposed metabolic imbalances, leading to improved crop performance. Phytohormone-producing microbes increase rice drought tolerance by improving root traits that are key to overcome drought conditions. The promotion of rice root and shoot growth under drought is regulated, among other factors, by the IAA-producing rhizobacterium *Streptomyces mutabilis*, resulting in increased soil water uptake and dry matter production (Suralta et al. 2018). The fungal endophyte *Arthrinium phaeospermum*-mediated drought tolerance in rice is ascribed to the boosted root architecture which is suggested to be influenced by GAs and IAA (Heutinck 2017). Likewise, the IAA-producing Class 2 endophytes improve rice salt tolerance by increasing the biomass of roots through preferential resource allocation (Redman et al. 2011), and the ability to enhance photosynthetic efficiency (Woodward et al. 2012). Another major microbial metabolic activity improving plant tolerance against various abiotic stresses is linked to the production of ACCD which lowers stress-induced ethylene levels (Glick 2004). Several endophytic bacteria improve rice physio-biochemical and growth performance under drought and salt stress conditions through their ACCD activity. Through this mechanism, *Streptomyces* sp. GMKU 336 improves rice growth, chlorophyll- and water-content, ROS scavenging, osmoprotection, and K^+^/Na^+^ ratio under salt stress (Jaemsaeng et al. 2018). An increase in Ca^2+^-content in the inoculated salt-stressed rice suggests that the endophyte also modulates calcium signalling network to help rice tolerate salt stress. Similarly, *Glutamicibacter* sp. YD01 alleviates adverse effects of salt stress on different physio-biochemical aspects of rice, including seed germination, chlorophyll- and water-content, photosynthesis capacity, ion-homeostasis, ROS accumulation, and antioxidation (Ji et al. 2020). Such activity has been attributed to the endophyte ability to modulate ACC content and ACC oxidase activity, again leading to decreased ethylene production. As residual ethylene can compromise plant growth under stress conditions, focus has been directed to improve the ACCD trait of endophytes and other microbes. By expressing ACCD enzyme on the cell surface of endophytes *Enterobacter* sp. E5 and *Kosakonia* sp. S1, making it more readily available for plant-produced ACC, inoculated rice sprouts showed up to 21% increase in ACCD activity over the wild-type and improved growth and salt tolerance (Liu et al. 2017b). Recently, the application of engineered endophyte *Bradyrhizobium* strain SUTN9-2 (showing increased ACCD activity) has been demonstrated to enhance rice performance under water deficit by reducing membrane damage and chlorophyll degradation, and simultaneously improving leaf relative water content (RWC), recovery rates, and crop yield in field conditions (Sarapat et al. 2020). Moreover, the use of *Azoarcus* sp. CIB, engineered for increased activity of ACCD, reduced ROS-induced damage under cadmium stress (Fernández-Llamosas et al. 2020). *Streptomyces mutabilis* also improves rice root architecture under drought conditions, which is attributed to its ACCD- and phosphatase-producing ability (Suralta et al. 2018). Other studies have also reported similar effect of ACCD-producing microbes (Arunthavasu et al. 2019; Pang et al. 2020; Kumar et al. 2021). Engineering endophytes or other PGP microbes for ACCD enzymatic activity therefore represents a way forward to ultimately boost rice production.

The production of EPS represents another crucial mechanism through which endophytes and other PGP bacteria promote plant growth under abiotic stresses, especially salt stress (Mokrani et al. 2020). EPS are high molecular weight complex microbial secretory polymers consisting of polysaccharides, proteins and small proportions of non-carbohydrate substituents including DNA, lipids, humic acids, and uronic acids (Schmid et al. 2015). The ionisable functional groups and substituents confer EPS an overall negative charge which favors binding of EPS to positively charged ions (Gupta and Diwan 2017). It has been shown that EPS-secreting endophytes alleviate salt toxicity and promote plant growth under salt stress by reducing Na^+^ absorption of plants (Ashraf et al. 2004). EPS also play a vital role in bacterial biofilm formation, in maintaining its functional and structural integrity and facilitate bacterial flocculation and colonization on root surfaces, ultimately promoting stress tolerance of the host plant (Shultana et al. 2020a). Several endophytes and rhizobacteria mitigate adverse effects of soil salinity on rice growth by secreting EPS. The endophyte *Pantoea alhagi* NX-11 secretes EPS enhancing rice shoot and root growth, fresh weight, and chlorophyll-content under salt stress by improving K^+^/Na^+^ ratio and osmotic balance and by reducing the salt-induced membrane damage (Sun et al. 2020). *Holomonas* rhizobacteria also produce EPS, contributing to alleviate salt and arsenic stress in rice (Mukherjee et al. 2019). The EPS-mediated biosorption of Na^+^ and arsenic, phosphate solubilization, and higher nutrient uptake increase seed germination and growth vigor of inoculated rice seedlings under these stresses. The EPS and biofilm-producing salt-tolerant rhizobacterial strain UPMRB9 (*Bacillus tequilensis*) adsorbs Na^+^ and promotes nutrient uptake, photosynthesis, transpiration and stomatal conductance, leading to higher filled-grain number, weight and the yield under salt stress (Shultana et al. 2020b). In another study, UPMRB9 was shown to improve rice physiological performance by increasing chlorophyll-content and RWC and decreasing electrolyte leakage and Na^+^/K^+^ ratio (Shultana et al. 2020a). These beneficial effects were attributed to bacterial EPS production and biofilm formation, demonstrating the existence of common mechanisms to enhance salt tolerance of rice. In support of this, EPS and biofilms produced by endophytes were also implicated in rice cold and drought tolerance (Kakar et al. 2016).

Endophytes also produce pigments and other antioxidative metabolites benefitting rice under abiotic stress conditions. The dark septate endophytic fungi from wild rice promote rice growth and biomass production under water deficit conditions and reduce the effects of water deficit-induced oxidative stress in rice by scavenging ROS (Dos Santos et al. 2017). The protective effects of these fungi against oxidative stress primarily owes to the pigment melanin present in their hyphae and microsclerotia (Barrow 2003). Moreover, the inoculation of pre-germinated rice seeds with thermotolerant fungal endophytes, isolated from desert-adapted plants, confers heat and drought tolerance at the early seedling stage of rice; with *Chaetomium* sp. significantly increasing survival and shoot and root growth under heat stress, while as *Aspergillus* sp. maintaining markedly high rate of root growth under drought (Sangamesh et al. 2018). The study suggests that *Aspergillus*-mediated protection of rice seedlings from stressful conditions is due to the presence of melanin in this fungus. The antioxidative potential of endophytic fungi is due to metabolites which possess extensive physiological activities. *Aspergillus fumigatus* SG-17 from riparian plants alleviates drought-induced oxidative stress in rice by producing (Z)-N-(4-hydroxystyryl) formamide (NFA), an analogue of coumarin with very high antioxidant activity, and by increasing their RWC (Qin et al. 2019b). SG-17-produced NFA regulates the oxidative pathway during drought-stress affecting the synthesis of ROS through the increase of antioxidants and heat shock proteins, and at the same time, inhibiting the activity of NADPH oxidases. The diazotropic endophytes from eudicots (poplar and willow) promote rice plant stature, tiller number and biomass, under nitrogen-limiting conditions by *in planta* N-fixation (Kandel et al. 2015). The larger population of these diazotrophic endophytes in roots supports rice root growth for improved water and nutrient uptake.

Other studies have demonstrated the beneficial effects of endophytes on rice growth and physiological performance under abiotic stress conditions without reporting on their metabolic activities, highlighting the need to expand this fundamental research. *Piriformospora indica* is an important endophytic filamentous fungus which symbiotically interacts with almost every crop species conferring protection from the adverse environmental conditions (Gill et al. 2016). Its large network of hyphae allows it to penetrate deep into soil and to transfer water and minerals (P and Zn) to inoculated rice under osmotic stress (Saddique et al. 2018). This symbiotic association also enhances chlorophyll fluorescence, Fv/Fm, proline accumulation, and total antioxidant capacity in osmotically stressed rice leaves. The uptake of P may account for an increase in biomass of the inoculated rice plants under osmotic stress because P promotes root growth and consequently water uptake and optimum leaf RWC during drought (Tariq et al. 2017). Increased concentration of Zn may account for the higher antioxidative capacity of inoculated rice, as Zn is an integral part of antioxidative enzymes such as SOD and CAT (Cakmak 2000). Under drought conditions, inoculation of rice with this endophyte enhanced plant photosynthesis, biomass and antioxidative potential, and reduced leaf wilting by diminishing the water loss through the promotion of stomata closure (Tsai et al. 2020). Under salt stress, *P. indica*-inoculated rice exhibit increased growth rate and biomass, higher photosynthetic pigment- and proline-content (Jogawat et al. 2013). These studies so far demonstrate that *P. indica* mitigates the abiotic stress-imposed damages in rice preferably by promoting the stress tolerance mechanisms such as ROS scavenging, osmotic balance, and protection of photosynthetic machinery.

An endophyte (EF0801) from a halophytic plant (*Suaeda salsa*) attenuates the toxicity induced by Na_2_CO_3_ (Bu et al. 2012) and Pb stress (Li et al. 2012) by triggering an antistress response, which includes the improvement of WUE, photosynthesis and of the antioxidant system, through increased production of photosynthetic pigments and high antioxidant enzyme activity. Both studies suggested that the stress tolerance in the EF0801-inoculated rice is due to the improved photosynthetic efficiency related to higher carotenoid and antioxidant enzyme content. However, both studies reported compromised root growth and biomass in contrast to other studies. Whether this is the result of the plant defence strategy, as suggested by the authors, remains to be demonstrated.

High atmospheric CO_2_ concentrations contribute to the global climate change, detrimentally affecting C3 photosynthesis and crop yield (Bhargava and Mitra 2021). Endophytes can be an innovative and sustainable solution to this problem. Salicaceae endophytes hold promise in mitigating the elevated CO_2_-dependent impairment of rice photosynthetic pathway, particularly under limited water supply (Rho et al. 2020). These endophytes display beneficial effects on both the demand (upregulating the rate of electron transport and production of NADPH and ATP during the light reaction) and supply sides (inducing leaf biophysical and biochemical barriers to hinder CO_2_ diffusion) (Rho et al. 2020).

PGP bacteria (PGPB) have been commonly isolated from roots or rhizosphere and reintroduced in plants by inoculation. In contrast, endophytic *Enterobacter* sp. SE-5 was isolated from rice sprouts and inoculated in seeds during germination (Liu et al. 2020b). Interestingly, the endophyte was found to be inheritable and systemically transmitting to the different organs of mature rice plants and promoting an increase in plant biomass under salt and Cd stresses. Therefore, the inoculation with sprout-endophytes can be used as an alternative feasible approach to those employing bacteria from roots or rhizosphere to promote plant growth under stressful conditions.

The combination of different endophytic and rhizospheric bacteria has been tested to identify possible synergies of action and added benefit for the plant. Rice inoculated with a consortium of *Pseudomonas pseudoalcaligenes* and *Bacillus pumilus* exhibit better response to salinity by accumulating higher levels of glycine-betaine-like quaternary ammonium compounds and biomass (Jha and Subramanian 2011). The authors suggested that glycine-betaine may increase Na^+^ flux from cytoplasm to vacuole, maintain homeostasis, and stabilize the oxygen evolution from PSII. Similarly, the application of salt-tolerant *Bacillus subtilis* in combination with farmyard manure was shown to improve rice root and shoot biomass in saline soils (Bhambure et al. 2018). Thus, the synergistic action of either different PGPB, or the mixture of PGPB with other organic amendments can overcome the adverse effects of salt stress in rice by regulating its growth and physiological performance. Hence, the use of these consortia could also be considered for alleviating other abiotic stresses in rice.

## Regulation of rice gene regulatory networks under abiotic stress conditions by endophytes: spotlight on salt stress response

The regulation of plant growth and physiological traits by endophytes and other PGP microbes under abiotic stresses is a complex phenomenon, which involves also extensive transcriptional reprogramming. The identification of the target genes can increase the understanding of the molecular mechanisms underlying plant stress responses and those underlying the plant-microbe interactions, with the goal to engineering stress-tolerant crops.

**Fig. 1:**
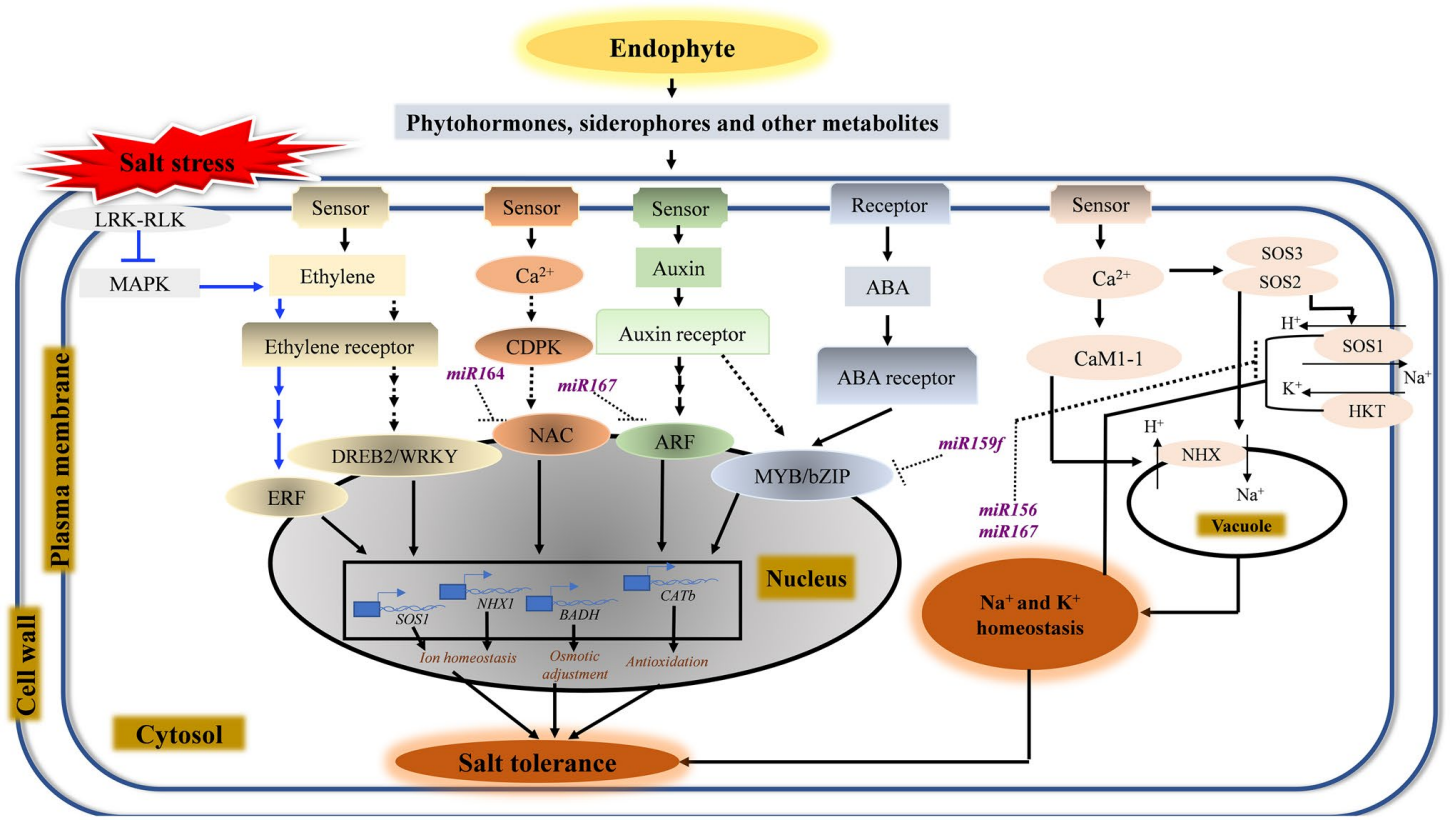
Molecular mechanisms regulating endophyte-mediated rice responses to salt stress. The salt stress signal is perceived by various signal transduction sensors transducing the signal along different pathways into the cell. These sensors include membrane-associated kinases, G-protein coupled receptors, glutamate receptor-like channels, calmodulin binding receptors, cyclic nucleotide-gated channels, and Ca^2+^ channel opening (Jamla and Archak 2019). Endophytes target different components in this signalling system during salt stress in the rice cell. They modulate either phytohormone- such as auxin, ethylene and abscisic acid (ABA) (Liu et al. 2017b; Khan et al. 2020; Sultana et al. 2020), mitogen-activated protein kinase (MAPK)-, or Ca^2+^-signalling (through calcium-dependent protein kinases (CDPK)) pathways (Jaemsaeng et al. 2018; Chauhan et al. 2019), which transduce the signal into the cell to modulate the expression of transcription factors (TFs) such as ethylene responsive factor (ERF), dehydration-responsive element-binding 2 (DREB2), NAC (NAM, ATAF and CUC), auxin response factor (ARF), and myeloblastosis (MYB). These TFs in turn induce salt-responsive genes (for each case, only representative genes are shown) such as *Salt Overly Sensitive 1* (*SOS1*) and *Sodium/Hydrogen eXchanger 1* (*NHX1*) for ion-homeostasis, *Betaine Aldehyde Dehydrogenase* (*BADH*) for osmotic adjustment, and *CATALASE b* (*CATb*) for antioxidation (Nautiyal et al. 2013; Ji et al. 2020). They can also target Ca^2+^-signalling pathway (through calmodulin, CaM1-1) leading to the SOS pathway-mediated ion-homeostasis in the rice cell by activating plasma membrane-bound SOS1 and vacuolar NHX1 (Jaemsaeng et al. 2018) proteins. Endophytes also induce the expression of various salt-responsive miRNAs which can regulate these TFs and structural genes like *SOS1* and *high-affinity K*^*+*^ *transporter 1* (*HKT1*) (Kord et al. 2019), supporting the existence of crosstalk between signalling pathways, conferring enhanced salt tolerance in rice. Dashed arrows indicate possible mechanisms. Blue arrows along the pathway from MAPK through ERF indicate endophyte-mediated pathway downregulation

Gene expression studies in endophyte-inoculated rice have identified molecular mechanisms associated with increased endogenous production of metabolites in response to different abiotic stresses. Auxin- and ACCD-producing *B. amyloliquefaciens* NBRISN13 (SN13) upregulates the expression of *nicotinamide adenine dinucleotide phosphate*-*malic enzyme 2* (*NADP-Me2*), *ethylene-responsive element binding protein* (*EREBP*), *salt overly sensitive 1* (*SOS1*), *betaine aldehyde dehydrogenase* (*BADH*), *somatic embryogenesis receptor kinase 1* (*SERK1*) and *catalase* genes in rice under salt stress conditions, which linked the bacteria’s ability to enhance salt tolerance in rice to processes including osmotolerance, malate degradation, ion-homeostasis, antioxidation, and nutrient-uptake (Nautiyal et al. 2013). These bacteria also stimulate the enrichment of osmoprotectant-utilizing microbial populations providing osmoadaptation to the inoculated plants. SN13 also alters the expression of genes for photosynthesis, hormone signalling, cell-wall and lipid metabolism (Chauhan et al. 2019). The endophyte-triggered signalling is transduced by calcium-dependent protein kinases (CDPKs) and mitogen-activated protein kinases (MAPKs) to the nucleus and other organelles where, through different types of transcription factors [NAC/MYB/bHLH/AP2-ERF/GAI (gibberellic-acid insensitive), RGA (repressor of GAI), and SCR (scarecrow) (GRAS)], the expression of genes associated with phytohormone signalling, and the adjustment of ionic and osmotic balance is enhanced for improved salt tolerance (Fig. 1). Treatment of rice with the combination of IAA- and siderophore-producing *B. amyloliquefaciens* and *B. laterosporus* improves resistance to drought and cold stress by inducing the expression of genes such as *OsMYB3R-2*, *dehydration-responsive element-binding protein 1 A* (*OsDREB1A*), *OsCDPK13*, and *drought-induced LTP* (*OsDIL*) (Kakar et al. 2016). The bacteria may be targeting the stress-triggered calcium signalling via OsCDPK13 to transduce the signal for activating the expression of NAC-domain containing regulatory proteins OsMYB3R-2 and OsDREB1A that could, in turn, regulate the expression of *OsDIL* which has a role in depositing water-proof lipid on plant surfaces to limit water loss. Hence, targeting primarily regulator genes might be a key strategy employed by endophytes to activate the plant stress responses in a robust way. Some IAA- and GA-producing endophytic bacterial strains, increase the expression of *OsYUCCA1* (flavin monooxygenase) and *PIN-FORMED 1* (*OsPIN1*, an auxin efflux carrier) genes in rice subjected to salt stress (Khan et al. 2020). Since *OsYUCCA1* is involved in auxin biosynthesis and *OsPIN1* facilitates auxin efflux from cells, the endophytes may systematically activate rice auxin signalling to promote root growth, enabling plants to uptake more nutrients and water under salt stress. The mechanisms through which the hormones produced by the microbes alter the expression of stress-responsive genes in rice remains to be demonstrated. The promotion of rice salt tolerance by ACCD-producing bacteria includes the modulation of key salinity-related genes. The endophytic *Streptomyces* sp. GMKU 336 enhances rice salt tolerance by repressing ethylene pathway genes, such as ACC synthase (*ACS1*) and ACC oxidase (*ACO1*), to reduce the ethylene-content and the associated stress (Jaemsaeng et al. 2018). The endophyte also mediates osmotic adjustment, ion-homeostasis and ROS scavenging by upregulating *BADH1*, calmodulin (*Cam1-1*), Na^+^ transporters (*sodium/hydrogen exchanger 1* (*NHX1*) and *SOS1*), and antioxidant enzymes (*CuZn-SOD1* and *CATb*), and by downregulating *MAPK5* (Jaemsaeng et al. 2018). Therefore, the endophyte GMKU 336 seems to enhance salt tolerance of rice through at least three different mechanisms (Fig. 1): (i) the activation of the calcium signalling network by increasing the intracellular concentration of Ca^2+^ which binds Cam1-1 to regulate downstream cellular processes, such as ion-homeostasis through SOS pathway (*SOS1*, *NHX1*) and ROS scavenging through antioxidant defence pathway (*CuZn-SOD1*, *CATb*); (ii) further contribution to ROS scavenging by downregulating *MAPK5* and ethylene signalling, because salinity-triggered MAPK cascade enhances ethylene production to activate ethylene signalling, which, in turn, promotes growth inhibition through ROS-induced damage (Steffens 2014); (iii) increasing proline- and glycine betaine-mediated osmotolerance by upregulating *OsBADH1*. Another ACCD-producing halotolerant bacterial strain, *Glutamicibacter* sp. YD01 increases rice salt tolerance in a way similar to GMKU 336, i.e., by modulating ethylene signalling, ion-homeostasis, and antioxidation (Ji et al. 2020). However, YD01 may accomplish these processes by targeting different sets of genes, namely increased K^+^/Na^+^ ratio by upregulation of *OsHKT1*, increased antioxidation by upregulation of *peroxidase 1* (*OsPOX1*), *iron-SOD* (*OsFeSOD*) and *glutathione reductase 2* (*OsGR2*), and repressed ethylene signalling by downregulation of *OsERF1* (Ji et al. 2020). Moreover, the expression of other salt-responsive genes *OsDREB2A* and *OsWRKY11* (tryptophan-arginine-lysine-tyrosine) was also upregulated. In conclusion, the two bacterial strains regulate salt tolerance in rice by modulating the expression of different sets of genes and possibly through different signalling pathways (Fig. 1), which might be due to the metabolic activities of the bacteria other than ACCD production. The EPS-producing endophyte *Pantoea alhagi* NX-11 mediates salt stress-induced damage in rice by upregulating the proline biosynthesis genes *pyrroline-5-carboxylate synthase* (*OsP5CS*) and *pyrroline-5-carboxylate reductase* (*OsP5CR*), and downregulating the proline catabolism gene *pyrroline-5-carboxylate dehydrogenase* (*OsP5CDh*). Proline thus mitigates salt stress by removing ROS and enhancing osmoprotection (Sun et al. 2020). *Bacillus aryabhattai* with multiple growth-promoting (nitrogen fixation, phosphate solubilization and IAA production) and EPS-producing activities, causes an increase in the expression of salt-responsive genes *BZ8* (ABA-responsive bZIP protein; osmotic signalling), *GIG* (GIGANTEA; ROS scavenging), and *NHX1* and *SOS1* (ion-homeostasis) in rice under saline conditions (Sultana et al. 2020), which could mitigate salt stress in rice by enhancing osmoprotection, antioxidation, and K^+^/Na^+^ ratio (Fig. 1). Also, the induction of *BZ8* by the IAA-producing endophyte suggests a cross talk between IAA and ABA signalling pathways under salinity in the rice cell. In addition, salt-stressed rice plants colonized with *high-osmolarity glycerol 1* (*HOG1*)-knockdown endophytes, show reductions in biomass, shoot and root growth, chlorophyll- and proline-contents, which is associated with the altered regulation of salt tolerance-related genes including *serine/threonine-protein kinase* (*OsSTK*), *late embryogenesis abundant* (*OsLEA*), *zinc-finger protein* (*OsAP1*), *magnesium-protoporphyrin IX* (*OsMPIX*), *40 S ribosomal protein S27* (*Os40S27*), *stress-induced protein* (*OsSIP*), and *OsSOS1;* suggesting that the fungal *HOG1* is involved in the regulation of rice osmoregulatory and osmoadaptive genes under salt stress (Jogawat et al. 2016).

Growing evidence shows that post-transcriptional regulation of gene expression plays a key role in the alleviation of abiotic stresses in rice (Djami-Tchatchou et al. 2017; Ganie 2020; Ganie and Reddy 2021). MicroRNAs (miRNAs) are vital post-transcriptional regulators with key roles in rice response to abiotic stresses (Jeong and Green 2013; Ganie et al. 2016). The molecular mechanisms through which endophytes confer stress tolerance in rice also involve these regulatory molecules (Fig. 1). *P. indica*-inoculated and salt-stressed rice exhibit differential abundance of miRNAs regulating target genes involved in salt-responsive processes such as import of K^+^ into and export of Na^+^ out of root cells (miR156, miR167), ROS detoxification (unannotated novel miRNA), nutrient uptake (miR166), and auxin-mediated growth (miR167) (Kord et al. 2019). The endophyte affects the expression abundance of miRNAs that regulate the expression of transcription factors associated with salt tolerance, such as miR159f for *MYB*, miR164 for *OsNAC5*, and miR396 for *OsGRF.* It is evident therefore that endophytes can fine-tune multiple crucial downstream processes in response to salt stress by modulating the expression of regulatory genes via miRNAs (Fig. 1). *P. indica* also confers drought tolerance in rice by modulating the expression of miR159 and miR396, thereby potentially affecting water-saving capacity and growth by regulating MYB and GRF transcriptional factors (Mohsenifard et al. 2017). However, the expression of miR396 in the two studies is contrasting. Thus, the analysis of different gene targets for the differentially regulated miRNAs will further the understanding of endophyte-rice interactions to better rice stress tolerance. Alternative splicing is another vital post-transcriptional modulator of gene expression amplifying the proteome diversity and regulating various physiological processes essential for mounting responses to abiotic stresses (Laloum et al. 2018). Recent studies in rice have shown that alternative splicing is increased in response to abiotic stresses to rapidly reprogram gene expression that is optimal for plant survival under unfavorable growth conditions (reviewed by Ganie and Reddy 2021). The salt-tolerant endophytic fungus *Fusarium* sp. reduces alternative splicing in salt-sensitive rice (IR64) under salt stress, possibly by minimizing the stress perception by the inoculated-plants through other mechanisms and thus obviating the need for otherwise crucial stress-responsive process of alternative splicing (Sampangi-Ramaiah et al. 2019). Nonetheless, a reduction in alternative splicing events for crucial cellular processes such as peroxisome biogenesis and DNA replication genes was observed (Sampangi-Ramaiah et al. 2019). Endophytes such as *Fusarium* sp can also activate other mechanisms than alternative splicing to increase rice salt tolerance. Namely, increasing the intracellular Ca^2+^ concentration and by regulating genes for signal perception (leucine-rich repeat proteins, kinases) and transduction (calmodulin-binding proteins), transcription factors (bHLH, MYB, WRKY and bZIP), secondary metabolism, and oxidative stress scavenging (glutaredoxins) (Sampangi-Ramaiah et al. 2020). An interesting arbuscular mycorrhizal fungus (*Glomus intraradices*)-mediated molecular mechanism, regulates root water uptake in rice under cold stress, and involves trehalose as a signalling molecule (Liu et al. 2014). At low temperature, the expression levels of fungi aquaporin (*GintAQPF*) and rice aquaporin (*OsPIPs*) genes increase, with simultaneous upregulation of trehalose biosynthesis genes (*OsTPS1*, *OsTPS2*, *OsTPP1*) in the mycorrhizal rice roots, leading to increased production of trehalose. It was suggested that the fungus enhance rice cold tolerance through the accumulation of trehalose that in turn can induce fungal and host plant aquaporin genes to maintain a better water status in the mycorrhizal rice. The fungus *Trichoderma harzianum* also improves drought tolerance in rice by preventing dehydration and enhancing water relations through upregulating rice dehydrin (*DHN1*) and aquaporin (*AQU*) genes (Pandey et al. 2016). Therefore, targeting root hydraulic conductance and water uptake mechanisms by regulating the aquaporin and other related genes can be another potential strategy adopted by microbes to mitigate the stress associated with water-deficit in rice.

Cell wall structure remodeling is a crucial response of rice to abiotic stresses (Kao 2017; Ganie and Ahammed 2021). Inoculation with *P. indica* increases growth performance of salt-stressed rice by maintaining cell wall integrity (Nivedita et al. 2020). Under salt stress, the fungus activates signal transduction by upregulating receptor-like and wall-associated kinase genes (LOC_Os01g26300, LOC_Os01g57510) to enhance plant growth that ultimately relies on augmented phytohormone production (by upregulating genes such as LOC_Os11g04190, LOC_Os09g35870) and maintenance of cell wall integrity (by upregulating genes such as LOC_Os06g21410, LOC_Os05g01380, LOC_Os07g36750).

Overall, the above examples not only provide evidence for the plethora of molecular mechanisms that rice has evolved to cope with adverse environmental conditions but also provide the grounds for future biotechnological yet environmentally friendly and sustainable solutions.

## The effect of endophytes on rice disease resistance

Host immunity is dependent on the plant microbiome. The endophytic metabolites may interfere with pathogen growth and development by subtracting essential minerals, inhibiting virulence, or promoting the plant growth under pathogen stress (Fadiji and Babalola 2020). One of the key mechanisms governing bacterial pathogenicity is quorum sensing, which involves communication among bacteria (Helman and Chernin 2015). The interference with this communication system has been regarded as a highly desired target for controlling bacterial diseases of plants. The virulence of *Burkholderia glumae* (causing seedling- and grain-rot of rice) was attenuated by the engineered endophytic *Burkholderia* sp. KJ006 that disrupted the pathogen quorum sensing through a counterpart mechanism called quorum quenching (Cho et al. 2007). Toxoflavin is a major virulence factor of this pathogen which requires quorum sensing for its production and utilizes *N*-acyl homoserine lactones (AHL) as auto-inducers (Suzuki et al. 1998). The engineered endophyte producing the AHL-degrading enzyme *N*-acyl-homoserine lactonase, was thus found to inhibit the production of quorum-sensing signals by the pathogen and to restrain the disease development in rice (Cho et al. 2007) Spores of the endophyte *Streptomyces endus* OsiSh-2 cause hyphal swelling and growth suppression of *Magnathoporthe oryzae*, thereby reducing the severity of rice blast disease (Xu et al. 2017). Besides, growth inhibition of the blast fungus with the fermentation broth and cell-free filtrates of OsiSh-2 indicates the role of extracellular enzymes and active metabolites underlying the pathogen inhibition. The endophyte produces cellulase, protease and gelatinase (can hydrolyze fungal cellulose and inhibit adhesion of fungal spores to plant surface), siderophores (can cause fungal death by starvation), IAA and ACCD (can promote plant growth), and importantly, nigercin antibiotic that suppressed the growth of the blast fungus (Xu et al. 2017). The *S. endus* OsiSh-2 strain was later labelled as *S. hygroscopicus* by NCBI. In two later studies, more details on the antagonistic activity of OsiSh-2 metabolites towards *M. oryzae*, which could be suppressing the blast disease in rice, was demonstrated. Zeng et al. (2018) showed that the ability of OsiSh-2 to produce high levels of siderophores, that is associated with the presence of siderophore biosynthetic gene clusters, increases the competition with the blast pathogen for iron and leading ultimately to its starvation. Later, Xu et al. (2019) showed that OsiSh-2 reduced rice blast disease under both greenhouse and field conditions and demonstrated that the endophyte severs the integrity of cell wall and cell membrane and impairs the TCA cycle in the blast fungal mitochondria. Additionally, the bacterial seed endophyte *Sphingomonas melonis* confers rice resistance to seed-borne pathogen *Burkholderia plantarii* by producing anthranilic acid that suppresses the pathogen by interfering with its sigma factor RpoS (Matsumoto et al. 2021).

There are reports on the biocontrol potential of endophytes against different rice bacterial and fungal pathogens *in vitro* (Nagarajkumar et al. 2004; Tian et al. 2004; Etesami et al. 2019; Ibrahim et al. 2019; Khaskheli et al. 2020) that hold potential to mitigate biotic stress response. Ibrahim et al. (2019) suppressed rice pathogenic bacteria, with the aid of silver nanoparticles and the endophyte *Bacillus siamensis* strain C1. The growth, biofilm formation and swimming motility of two rice bacterial pathogens *Xoo* strain LND0005 and *Acidovorax oryzae* strain RS-1, were inhibited, indicating the potential of nanotechnology to augment the defensive capacity of endophytes and its promise to protect rice plants from pathogen attack.

## Mechanisms of endophyte-conferred disease resistance in rice

Stress imposed by biotic agents such as bacteria, fungi and insects constantly inflict serious damage to rice production with enormous yield penalties. Some of the molecular bases of rice resistance mechanisms have been elucidated, including disease resistance genes and QTLs, phytohormone signalling, and other signalling molecules (Fahad et al. 2014; Takatsuji 2014; Ke et al. 2017; Singh et al. 2018; Zhang et al. 2019; Jiang et al. 2020). Endophytes play an important role in mitigating biotic stress in rice by potentiating molecular and physiological responses to improve plant growth and development. For example, inoculation with endophyte *P. indica* suppresses the rice root herbivory by water weevil (*Lissorhoptrus oryzophilus*), and enhances plant tolerance to the insect by increasing shoot and root growth, which is in turn mediated by GA and JAs signalling pathways (Cosme et al. 2016). It was demonstrated that the rice infested below ground with insect larvae, produced high level of JAs (12-oxophytodienoic acid, JA and jasmonoyl-isoleucine) in roots, which was enhanced by the JAs generated above ground by prior attack by adult leaf herbivory. The high JAs levels potentially suppress GA biosynthesis and cause accumulation of DELLA (aspartate-glutamate-leucine-leucine-alanine) proteins, leading to the inhibition of plant growth. To counter the pathogen attack, the endophyte-mediated activation of GA signalling in rice roots causes degradation of DELLA and inhibits JAs signalling, leading to increased plant growth and tolerance to root herbivory (Cosme et al. 2016). Priming with SN13 enhances rice immune response against *Rhizoctonia solani* by modulating ROS levels through production of ROS quenchers (arabitol, proline, mannitol and phospholipases), production of non-metabolizable rare sugars (turanose) to keep the immune system induced and compromise fungal growth; activation of defence response through MAPK signalling and production of defence-related alkaloids such as terpenes and quinazoline, and maintaining cell wall integrity (Srivastava et al. 2016). The reduced starch-content and the delayed formation of aerenchyma in parenchymal cells of SN13-inoculated rice suggest delayed apoptosis of these cells for enhanced stress tolerance, which is attributed to the high production of a hypoxia-responsive protein ENOD18 (early nodulin 18) in these plants (Srivastava et al. 2016). Although ROS scavenging is associated with increased plant biotic stress tolerance, increased production of ROS is also implicated in pathogen defence (Torres et al. 2006). High production of ROS in endophyte (*Pantoea*, *Pseudomonas* sp., *Paenibacillus* sp., *Chryseobacterium* sp., etc.)-colonized rice roots is also suggested to induce resistance against many fungal pathogens (*Fusarium oxysporum*, *Curvularia* sp. and *Alternaria* sp.) by possibly regulating defence-related genes (Verma et al. 2018).

## Endophyte induced systemic resistance (ISR)

**Fig. 2:**
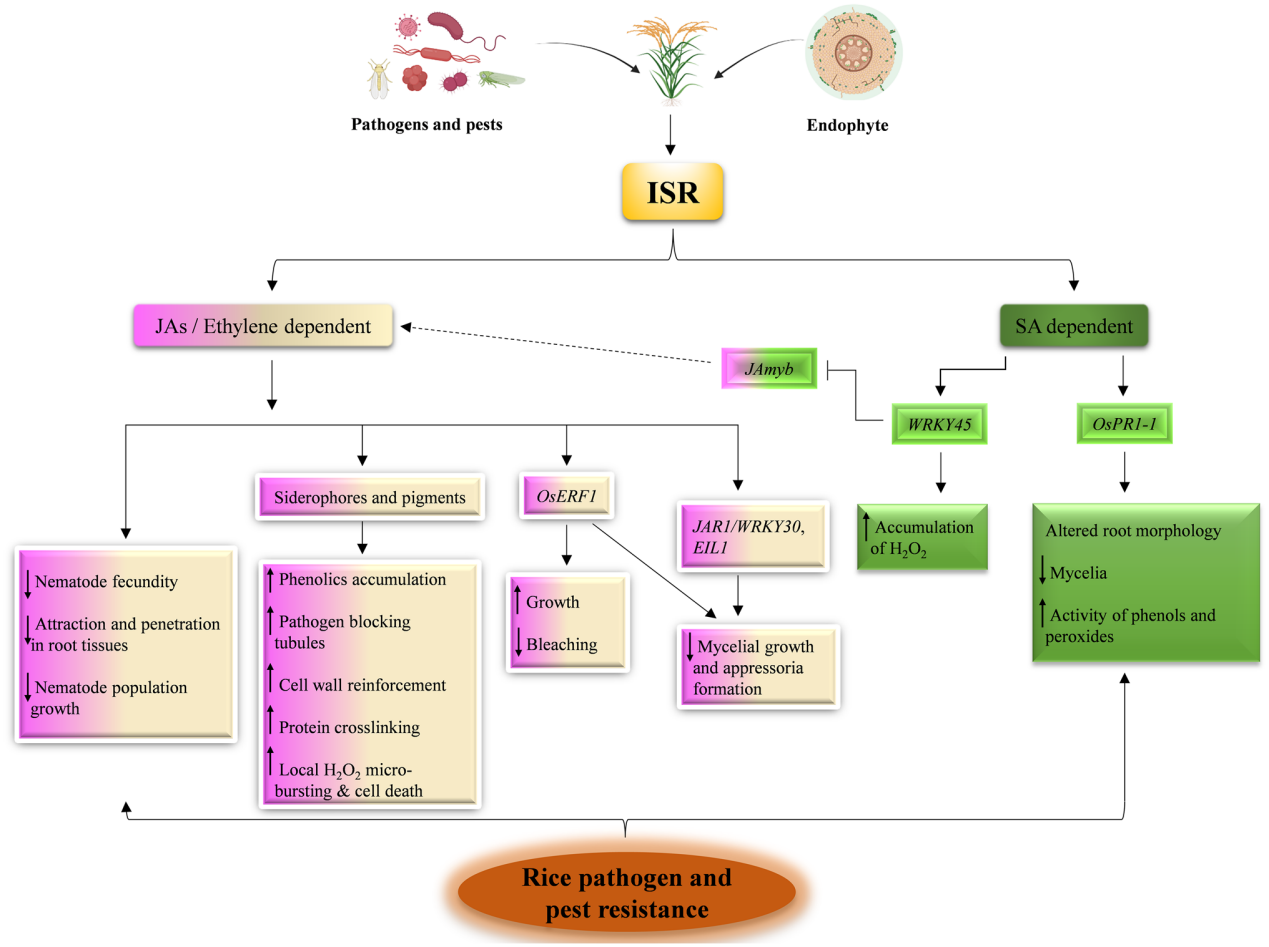
Endophyte-Induced systemic resistance (ISR) in rice. ISR conventionally depends on JAs and ethylene signalling. Endophytes, through production of siderophores such as pseudobactin and pigments like pyocyanin, induce robust cellular defences against pathogens (De Vleesschauwer et al. 2006, 2008); while through other unknown mechanisms, they impair development, fecundity and population size of rice pathogens (Le et al. 2016). They also promote plant growth but inhibit pathogen growth through regulating the expression of rice genes involved in ethylene signalling such as *ethylene responsive factor1* (*OsERF1*), *ETHYLENE INSENSITIVE 3-like 1* (*EIL1*), and JAs signalling such as *jasmonate resistant1* (*JAR1*), *tryptophan-arginine-lysine-tyrosine30* (*WRKY30*) (Spence et al. 2014). The nonconventional salicylic acid (SA)-dependent endophyte-ISR in rice can occur either through repressing JA-signalling pathway by downregulating *JAmyb* (Lee et al. 2001), or through *NON-EXPRESSOR of PR1* (*OsNPR1*)- and *OsWRKY45*-mediated signalling pathways (Su et al. 2013; Ashajyothi et al. 2020), leading to the inhibition of pathogen growth on account of H_2_O_2_ burst, altered plant morphology, and phenolics production

Besides direct antagonism, several endophytes and other PGP microbes can activate plant immunity through induced systemic resistance (ISR) that is effective upon future exposure to pathogens and enhancing pathogen resistance in the whole plant (Kloepper and Ryu 2006). This resistance mechanism (through priming) is generally mediated by JAs- and ethylene-dependent signalling pathways, and typically does not involve SA or *PATHOGENESIS RELATED* (*PR*) gene activation (Romera et al. 2019). However, there are exceptions to this rule and microbe-mediated ISR can also require SA and can be independent of JAs/ethylene, as discussed below. The ISR is a cost-effective mechanism because its fitness costs in plants are reduced in comparison to constitutively active defence (van Hulten et al. 2006). Microbiome-produced secondary metabolites play a crucial role in rice defence mechanisms (Fig. 2). *Pseudomonas fluorescens* WCS374r mounts ISR in rice against *M. oryzae* in a JAs-/ethylene-dependent but SA-independent manner, which is based on the priming effect of the siderophore pseudobactin and results in a robust cellular defence response that includes rapid accumulation of phenolics at pathogen entry sites, activation of structural defences like pathogen-blocking tubules, and hydrogen peroxide-mediated cell wall reinforcement and protein-cross linking (De Vleesschauwer et al. 2008). Similarly, redox-active pigment pyocyanin secreted by *P. aeruginosa* 7NSK2 triggers ISR against blast-causing *M. grisea* through local H_2_O_2_ microbursting on the root surface, thus priming the systemic naive shoot tissues for enhanced expression of hypersensitive response-like cell death to restrain the pathogen upon subsequent attack (De Vleesschauwer et al. 2006). The bacterial endophyte *Azospirillum* sp. B510 enhances resistance of rice to *M. oryzae* and *Xoo* independently of SA-dependent defence signalling (Yasuda et al. 2009). Recently, through gene expression and mutant analyses, Kusajima et al. (2018) elucidated the molecular mechanism behind B510-mediated blast resistance in rice, showing that the endophyte activated the expression of ethylene response factor gene (*OsERF1*), and that therefore ISR occurs through ethylene-signalling. Similarly, a rhizospheric *Pseudomonas* isolate, EA105, suppresses mycelial growth and appressorium formation of blast fungus in rice by triggering both JAs- and ethylene-mediated ISR through the expression of JAs- (*jasmonate resistant 1* (*JARI*) and *WRKY30*) and ethylene- [*ETHYLENE INSENSITIVE 3-like 1* (*EIL1*) and *ERF1*] signalling pathway genes (Spence et al. 2014). Taken together, these studies support the notion that different PGPB employ distinct strategies to mount ISR in rice (Fig. 2) and indicate that rice is equipped with multiple blast resistance mechanisms. Several studies on endophyte- and other PGP microbe-conferred disease resistance through induction of ISR in rice, have focused on defence-related enzymes (phenylalanine ammonia-lyase, peroxidase and polyphenol oxidase) and total phenol content (Jha and Subramanian 2011; Nagendran et al. 2014; Rangjaroen et al. 2019; Jain et al. 2020; Saikia and Bora 2020). The production of these enzymes and phenolsby consortia of these microbes induced better disease resistance than when they are applied individually (Jain et al. 2020; Saikia and Bora 2020) and endophytic bacteria perform better than rhizobacteria in defending rice (Jha and Subramanian 2011). Further, the complex genetic environment and the quality (determined by carbon, nitrogen, and defensive compounds) of the host plant are crucial factors affecting the fecundity potential and the reproductive success of herbivore insects (Awmack and Leather 2002; Kliebenstein 2018). In rice, the endophyte *Fusarium moniliforme* Fe14, cumulatively inhibits the long-term population development of root-knot nematode *Meloidogyne graminicola* by decreasing the nematode attraction and penetration into rice roots, increasing the male-female ratio, and by delaying the development of females inside the root tissue (Le et al. 2016). Through split-root assays, the endophyte was shown to induce systemic resistance against the nematode in rice. Therefore, the endophyte might induce a basal response mechanism in rice contributing to the improved quality of the plant, and leading to the impaired development, fecundity, and reproduction of the nematode during subsequent attack (Fig. 2). Despite these findings, endophyte-ISR in rice can also require SA accumulation and can thus occur through SA-dependent but JAs-/ethylene-independent signalling pathway (Fig. 2). For example, the inoculation of rice with endophyte *Harpora oryzae* reduces the expression of the JAs-signalling pathway gene *JAmyb* (Lee et al. 2001). The endophyte protects rice from *M. oryzae* through the accumulation of H_2_O_2_ and antioxidants, and through the SA signalling pathway which depends on *OsWRKY45* (Su et al. 2013). Hence, this endophyte induces both the local (by ROS) and systemic (by SA signalling) resistance in rice against blast pathogen. The endophyte *Bacillus oryzicola* YC7007 protects rice from bakanae disease-causing *Fusarium fujikuroi* in a similar way: inducing local resistance by accumulating H_2_O_2_ at an early stage and the systemic resistance, however, via the JAs signalling pathway (Hossain et al. 2016). Another example of unusual SA-dependent systemic resistance activated in rice by an endophyte comes from the induced defence against *M. oryzae* by *Pseudomonas putida* BP25 (Ashajyothi et al. 2020). However, unlike Su et al. (2013) (where ISR occurs through OsWRKY45-dependent pathway), the BP25-mediated systemic resistance against rice blast possibly involves *NPR* (NON-EXPRESSOR of PR)-dependent pathway, as indicated by the high expression of *PR* gene *OsPR1-1* (Ashajyothi et al. 2020). As the SA signalling pathway in rice occurs through *OsNPR1*- and *OsWRKY45*-dependent subpathways (Shimono et al. 2007), the endophyte-mediated nonconventional SA-dependent systemic resistance in rice against blast infection can be activated through either of the signalling pathways (Fig. 2), which depends therefore on the type of endophytic species.

## Conclusions and outlook

Global climate change is happening, and it is therefore urgent and paramount to develop high-yielding and stress tolerant crop varieties without the need to use chemical treatments that exacerbate the situation. The impact of climate change on rice is huge and leads to significant losses in grain yield and quality. It is necessary, therefore, to develop new strategies to enhance rice stress tolerance and consequently yield.

This review has provided evidence that plant genotype, growth stage, the type of environmental stress, and their interaction contribute to restructuring the endophytic microbial communities. The enhanced stress resilience of tolerant genotypes can be attributed, at least in part, to the differences in diversity and enrichment of endophytes they harbour. Ultimately, the constituents of the stress-altered endophytic microbial communities contribute to rice survival under unfavourable environmental conditions, and the enrichment of certain stress-responsive taxa could expedite the recovery response of rice. This has exciting implications on stress mitigation strategies using endophytic microbiota. Detailed dissection of genetic and molecular mechanisms specific to rice-endophyte interactions integrated with information of their co-evolution are going to be fundamental for future manipulation. Engineering rice genetic pathways regulating microbiome homeostasis through state-of-the-art genomic technologies is a promising route to developing an improved beneficial microbiome, which can subsequently improve rice performance under unfavourable environments. Also important is to continue exploring the environmental influence on native endophytic communities inhabiting rice organs to aid sustainable development of inoculants with minimal impact on the environment.

## Data Availability

Not applicable.
